# Genetic engineering of porcine endothelial cell lines for evaluation of human-to-pig xenoreactive immune responses

**DOI:** 10.1038/s41598-021-92543-y

**Published:** 2021-06-23

**Authors:** Ping Li, Julia R. Walsh, Kevin Lopez, Abdulkadir Isidan, Wenjun Zhang, Angela M. Chen, William C. Goggins, Nancy G. Higgins, Jianyun Liu, Randy R. Brutkiewicz, Lester J. Smith, Hidetaka Hara, David K. C. Cooper, Burcin Ekser

**Affiliations:** 1 Division of Transplant Surgery, Department of Surgery, Indiana University School of Medicine, Indianapolis, IN USA; 2 Indiana University Health, Indianapolis, IN USA; 3 Department of Microbiology and Immunology, Indiana University School of Medicine, Indianapolis, IN USA; 4 Radiology and Imaging Sciences, Indiana University School of Medicine, Indianapolis, IN USA; 5 3D Bioprinting Core, Indiana University School of Medicine, Indianapolis, IN USA; 6 Xenotransplantation Program, Department of Surgery, University of Birmingham at Alabama, Birmingham, AL USA; 7 Present Address: Weldon School of Biomedical Engineering, West Lafayette, IN USA

**Keywords:** Innate immune cells, Transplant immunology, Genetic engineering

## Abstract

Xenotransplantation (cross-species transplantation) using genetically-engineered pig organs offers a potential solution to address persistent organ shortage. Current evaluation of porcine genetic modifications is to monitor the nonhuman primate immune response and survival after pig organ xenotransplantation. This measure is an essential step before clinical xenotransplantation trials, but it is time-consuming, costly, and inefficient with many variables. We developed an efficient approach to quickly examine human-to-pig xeno-immune responses in vitro. A porcine endothelial cell was characterized and immortalized for genetic modification. Five genes including *GGTA1*, *CMAH*, *β4galNT2*, *SLA-I α chain,* and *β2-microglobulin* that are responsible for the production of major xenoantigens (αGal, Neu5Gc, Sda, and SLA-I) were sequentially disrupted in immortalized porcine endothelial cells using CRISPR/Cas9 technology. The elimination of αGal, Neu5Gc, Sda, and SLA-I dramatically reduced the antigenicity of the porcine cells, though the cells still retained their ability to provoke human natural killer cell activation. In summary, evaluation of human immune responses to genetically modified porcine cells in vitro provides an efficient method to identify ideal combinations of genetic modifications for improving pig-to-human compatibility, which should accelerate the application of xenotransplantation to humans.

## Introduction

Pig-to-human xenotransplantation (XTx) offers a promising solution to address the persistent organ shortage for clinical transplantation^1^. However, incompatibilities between pig and human species result in destructive human immune responses and ultimate failure of pig tissue/organ grafts. Genetic modification of pigs is an essential approach to overcoming these obstacles^2^. Elimination of all three major carbohydrate xenoantigens αGal, Neu5Gc, and Sda by disruption of the α1,3-galactosyltransferase gene (*GGTA1*), cytidine monophospho-N-acetylneuraminic acid hydroxylase gene (*CMAH*), and β-1,4-N-acetyl-galactosaminyltransferase 2 gene (*β4galNT2*) as well as transgenic expression of human complement-regulatory proteins (e.g. CD46, CD55), human coagulation-regulatory proteins (e.g. thrombomodulin, endothelial cell protein C receptor), anti-inflammatory molecule human heme oxygenase-1 (HO-1), and human macrophage-inhibitory ligand (CD47) to modulate human immune response, brings XTx closer to reality^3^. Recently, breakthrough progress has been made in preclinical XTx which has resulted in extended survival of pig-to-nonhuman primate (NHP) kidney XTx for more than a year^4,5^ (rhesus macaque), orthotopic heart XTx for more than 6 months^6^ (baboon), and heterotopic heart XTx more than 3 years^7^ (baboon). These exciting preclinical results have demonstrated the great potential of XTx for clinical application in the near future. Recent advances in gene-editing tools have accelerated the process of engineering of porcine cells and the production of genetically modified (GM) pigs. We have successfully eliminated multiple xenoantigens simultaneously in a single-step transfection using CRISPR/Cas9 gene-editing tool^8–10^. Yet, the challenge remains on how to efficiently evaluate the human immune response induced by various genetic modifications, and to identify an ideal genetic combination.

Currently, pig-to-NHP XTx is the standard preclinical model, which monitors NHP recipient survival (as surrogates for humans) after receiving life-supporting pig tissue/organ transplants. Pig-to-NHP XTx is a long process involving generation of GM pigs, XTx surgeries, and post-transplant animal care. It usually takes more than a year to complete one study. In addition, the difference of antibody reactivity to *CMAH* deficient porcine cells between human and NHPs suggests the potential limitation of NHP models for examining specific genetic modification(s)^9^. Our previous studies demonstrated that the elimination of three carbohydrate xenoantigenic epitopes αGal, Neu5Gc, and Sda, dramatically reduces human antibody binding to porcine cells^9,11^. The triple-gene knockout (TKO) (*GGTA1*, *CMAH*, *and β4galNT2*) pig is regarded as an essential genetic background for clinical XTx trials. However, elimination of porcine Neu5Gc may have detrimental effects in pig-to-NHP preclinical XTx trials. Antibody-mediated xenograft rejection in baboons was associated with the absence of Neu5Gc in pig cells/organs. Li et al.^12^ reported that baboons have elevated antibody binding to TKO pig red blood cells (RBC) compared to the RBC from *GGTA1/ β4galNT2* double-knockout (DKO) pigs. A recent study of pig-to-baboon renal XTx revealed that the median survival of TKO pig kidneys (4 days) was significantly shorter than the survival of *GGTA1*-knockout (Gal-knockout, GTKO) kidneys (136 days)^13^. Both pigs and NHPs have *CMAH* gene encoding an enzyme that hydroxylates N-acetylneuraminic acid (Neu5Ac) to produce Neu5Gc^14^. Inactivation of the porcine *CMAH* gene results in unknown xenoantigen exposure, which most likely becomes the target of NHPs pre-existing antibodies and triggers antibody-mediated rejection in NHPs. Consequently, different GM pigs are required to generate for pre-clinical and clinical XTx trials, respectively, which is not ideal for timely clinical application of XTx. Other approaches to evaluate pig genetic modifications are ex vivo perfusion of a GM pig organ with human blood^15^ and in vitro analysis of the human immune response to PBMC or kidney fibroblasts from GM pigs^10,11,16^, which still need the physical production of GM pig(s). Therefore, establishment of novel in vitro systems will be helpful to accelerate the screening process of compatible genetic modifications.

Human antibody-mediated rejection can be managed by removal of major xenoantigens. However, the human anti-pig cellular response remains as a significant impediment to greater success in XTx. There is an urgent need to examine human humoral and cellular immune responses to GM porcine cells to efficiently identify suitable genetic modifications for XTx. A novel pig cell model system will be useful to examine human anti-pig cellular response directly.

Porcine endothelial cell line was chosen in this study due to the following reasons: (i) porcine endothelial cell activation and damage are the first episodes of xeno-rejection^17,18^; (ii) porcine endothelial cells not only express all three carbohydrate xenoantigens^9^, but also express SLA-II and elevated SLA-I molecules upon proinflammatory cytokine stimulation. Both SLA-I and SLA-II molecules have been identified as xenoantigens^16,19^; (iii). Porcine endothelial cells can also be activated by human proinflammatory cytokines such as TNF-α, IL-1β, and IL-4^20^; (iv) porcine endothelial cells express activating ligands for human lymphocyte-activating receptor NKG2D to trigger NK cell- and T cell-mediated cytotoxicity^21^. All these features make porcine endothelial cells a favorable in vitro model to evaluate human anti-pig humoral and cellular immune responses to various genetic modifications.

Establishment of an in vitro model system to evaluate various genetic modifications will provide valuable knowledge for clinical trials and accelerate the application of XTx in humans. Here, we describe the generation of porcine cell lines with multiple-gene modifications. Both the human antibody response and human innate immune response to these engineered porcine cells have been investigated.

## Results

### Immortalization of porcine liver-derived endothelial cells

**Figure 1:**
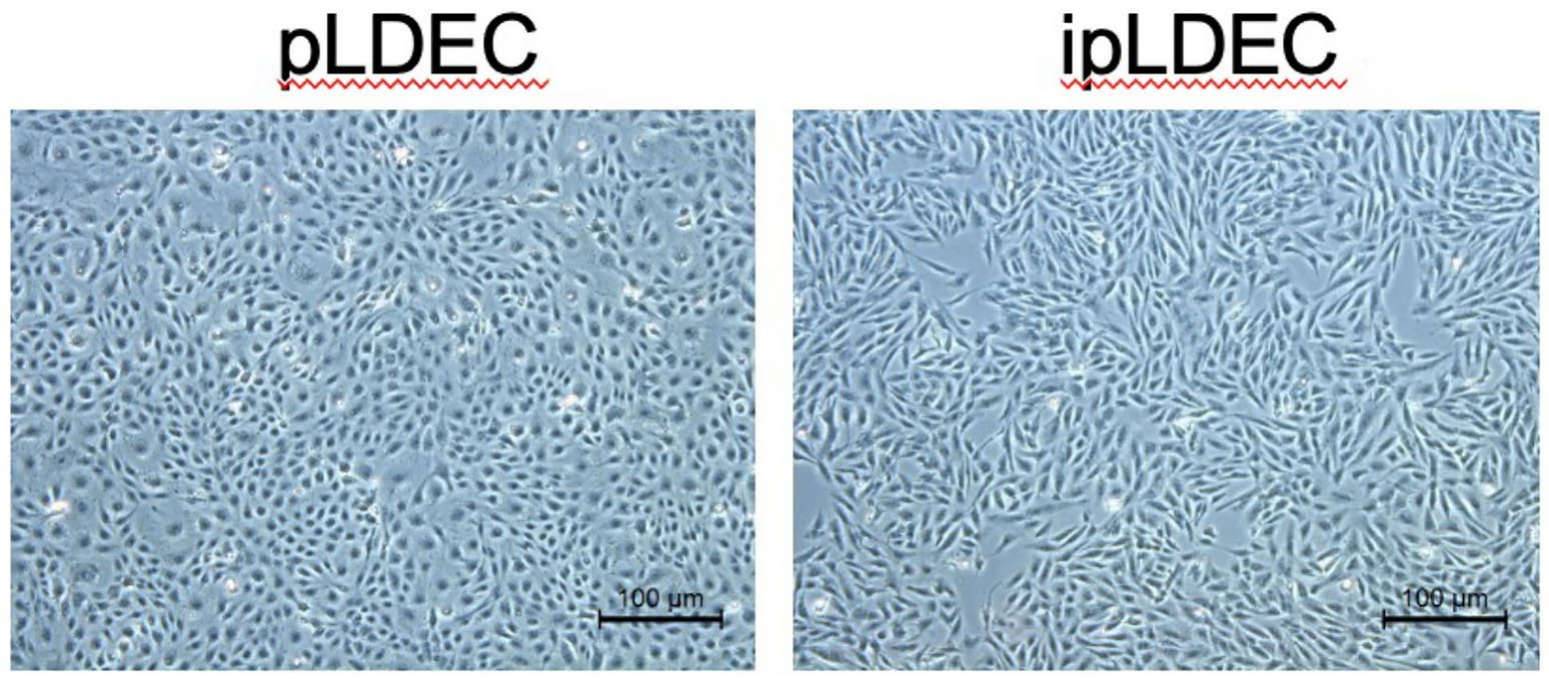
Morphology of primary pLDEC (passage 12) and WT ipLDEC (passage 25 after immortalization).

A primary porcine liver-derived cell (pLDEC) was isolated from a mature female crossbred domestic pig in a previous study^22^, which exhibited endothelial cell-like morphology. It had been previously used for multiple-gene modifications and various GM pig production, including *GGTA1/β4galNT2* DKO^4^, *GGTA1/CMAH/iGb3S* TKO^8^, *GGTA1/CMAH/ β4galNT2* TKO^9^, and *GGTA1/ASGR1* DKO^23^. This cell expresses blood type O^24^. In order to make various genetic modifications and to test the human immune response to these genetic modifications, pLDEC line (passage 12) was treated with lentivirus expressing the SV40 T antigen and hTERT to generate an immortalized wild-type (WT) pLDEC line (ipLDEC). Immortalization process did not alter WT ipLDEC (passage 25 after immortalization) morphology compared to primary pLDEC (passage 12) (Fig. 1).

### Establishing a five gene-knockout (5GKO; *GGTA1/CMAH/β4galNT2/SLA-I α chain/B2M*) ipLDEC line

**Figure 2:**
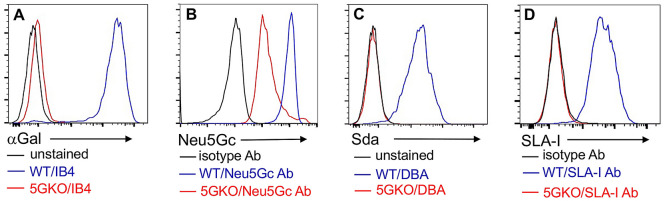
Phenotype of GM ipLDEC. Flow cytometric analysis of the absence of αGal (**A**), Sda (**C**), and SLA-I (**D**), and reduction of Neu5Gc (**B**) on ipLDEC (due to uptake of Neu5Gc glycan from Neu5Gc-rich FBS in culture medium).

**Figure 3:**
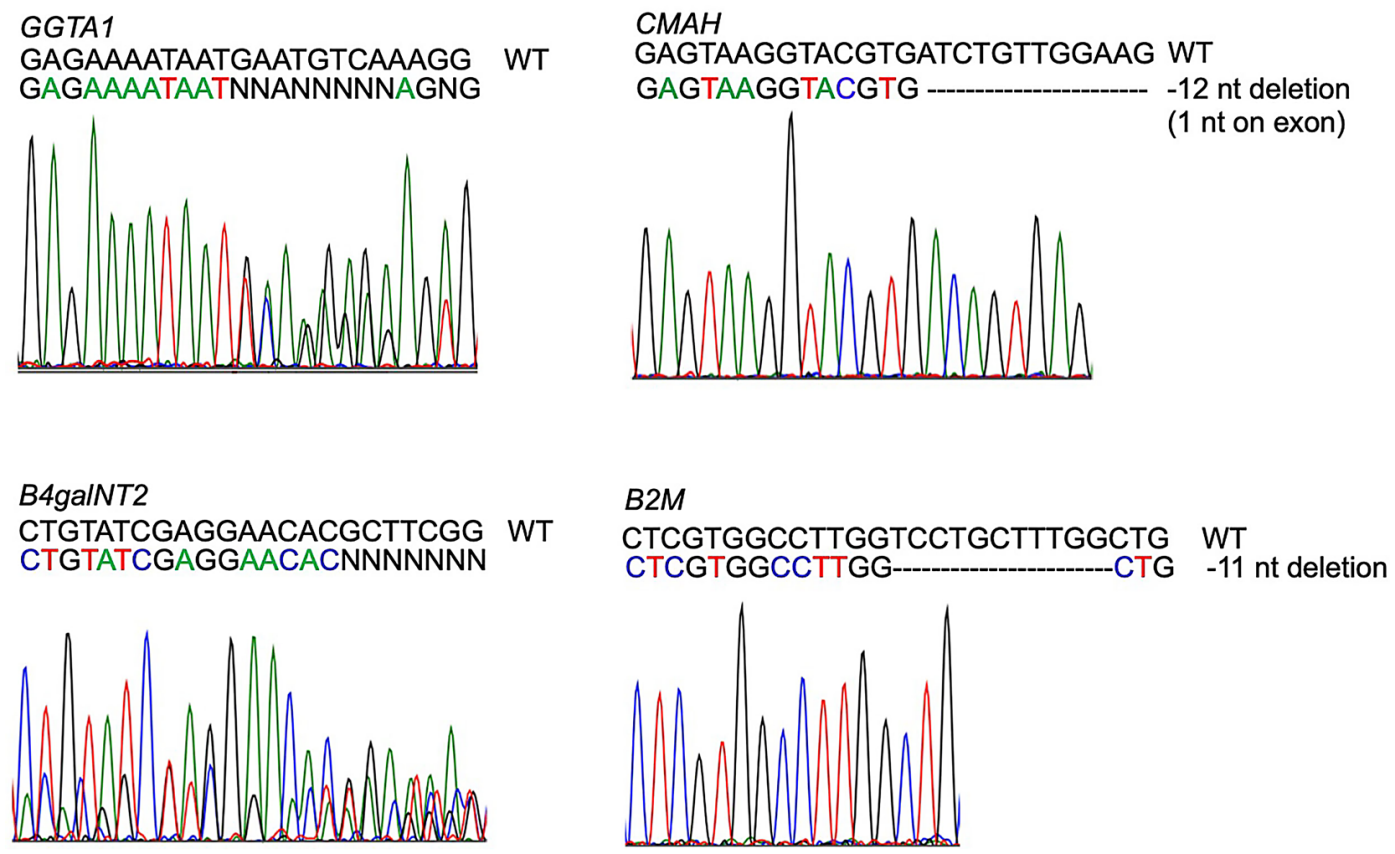
Genotype of GM ipLDEC. Each gRNA targeted region was amplified by PCR. Sanger sequencing revealed mutations in *GGTA1*, *CMAH*, *β4galNT2*, and *B2M* genes.

WT ipLDEC was co-transfected with CRISPR/Cas9 plasmids with guide-RNA (gRNA) targeted to *GGTA1*, *CMAH*, and *β4galNT2* genes (Table1), respectively. After sequential phenotypic selection, an ipLDEC line null for αGal, Neu5Gc, and Sda (TKO) was obtained (Fig. 2A–C). The gRNA targeted region of each gene was amplified by PCR using specific primers (Table 2). The mutations of *GGTA1, CMAH,* and *β4galNT2* genes were confirmed by Sanger sequencing (Fig. 3). Due to uptake and metabolic incorporation of the Neu5Gc glycan from Neu5Gc-rich FBS in culture media, some Neu5Gc was detected in TKO ipLDEC, but the mean fluorescent intensity (MFI) of Neu5Gc in TKO was decreased by 73% compared to the expression of Neu5Gc in WT ipLDEC (MFI: 28509 vs 105646) (Fig. 2B). This is a very common phenomenon when culturing *CMAH gene* deficient cells in medium with FBS. There were 12 nucleotides missing in *CMAH* genomic DNA (Fig. 3). Of which, the deletion of the first nucleotide in exon 4 resulted in a frame shift and the polypeptide terminates early at position 162. Alignment of the truncated CMAH polypeptide and the intact CMAH protein was analyzed using MacVector 18.0 software (MacVector, Apex, NC) (Supplementary Fig. S1). The truncated protein is unlikely to retain hydroxylase activity and to produce Neu5Gc.

**Table 1:** Sequences of gRNA target and gRNA oligos.

Gene	gRNA target	gRNA oligo
*GGTA1*	GAGAAAATAATGAATGTCAA	5′-CACCGAGAAAATAATGAATGTCAA-3′ 5′-AAACTTGACATTCATTATTTTCTC-3′
*CMAH*	GAGTAAGGTACGTGATCTGT	5′-CACCGAGTAAGGTACGTGATCTGT-3′ 5′-AAACACAGATCACGTACCTTACTC-3′
*β4galNT2*	CTGTATCGAGGAACACGCTT	5′-CACCGTGTATCGAGGAACACGCTT-3′ 5′-AAACAAGCGTGTTCCTCGATACAC-3′
*B2M*	CTCGTGGCCTTGGTCCTGCT	5′-CACCGTCGTGGCCTTGGTCCTGCT-3′ 5′-AAACAGCAGGACCAAGGCCACGAC-3′
*SLA-1,2,3* (1)	ATCATGTACGGCTGCGACGTG	5′-CACCGTCATGTACGGCTGCGACGT-3′ 5′-AAACACGTCGCAGCCGTACATGAC-3′
*SLA-1,2,3* (2)	ACTATTGGGATGAGGAGACGCGG	5′-CACCGCTATTGGGATGAGGAGACG-3′ 5′-AAACCGTCTCCTCATCCCAATAGC-3′

**Table 2:** PCR primers used to amplify gRNA targeting regions.

Gene	PCR primers	Amplicon (bp)
*GGTA1*	CCTTAGTATCCTTCCCAACCCAGAC GCTTTCTTTACGGTGTCAGTGAATCC	428
*CMAH*	CTTGGAGGTGATTTGAGTTGGG CATTTTCTTCGGAGTTGAGGGC	458
*β4galNT2*	CGCAAGTGACCAGACATCGTTC AAAGCCACAGGAGGAGCCAG	530
*B2M*	TCTTTCTAACCTGCTCGGGC CGATCTGAAGCTTACCCGCA	530

SLA-I molecules consist of polymorphic α chains and conserved β2-microglobulin (B2M). To completely remove SLA-I in porcine cells, we designed gRNA targeting both *α chains* and *B2M genes*. TKO ipLDEC line was transfected with plasmids containing two selected gRNAs targeting the common sequences in multiple alleles of *SLA-I α chains*. Disruption of *SLA-I α chains* led to the loss of SLA-I expression, so the mutant cells were selected by flow cytometry cell sorting (Fig. 2D).

This four-gene-knockout cell line (*GGTA1/CMAH/ β4galNT2/SLA-I α chain*) was transfected with the plasmid bearing gRNA targeting the *B2M* gene. *B2M* mutants were screened by PCR and Sanger sequencing, and a clone with 11 nucleotide-deletion was selected (Fig. 3). Thus, an ipLDEC line with 5-gene-knockout (5GKO; *GGTA1/CMAH/ β4galNT2/SLA-I α chain/B2M*) was successfully established.

### Immortalized pLDEC express endothelial cell markers and respond to cytokine stimulation

**Figure 4:**
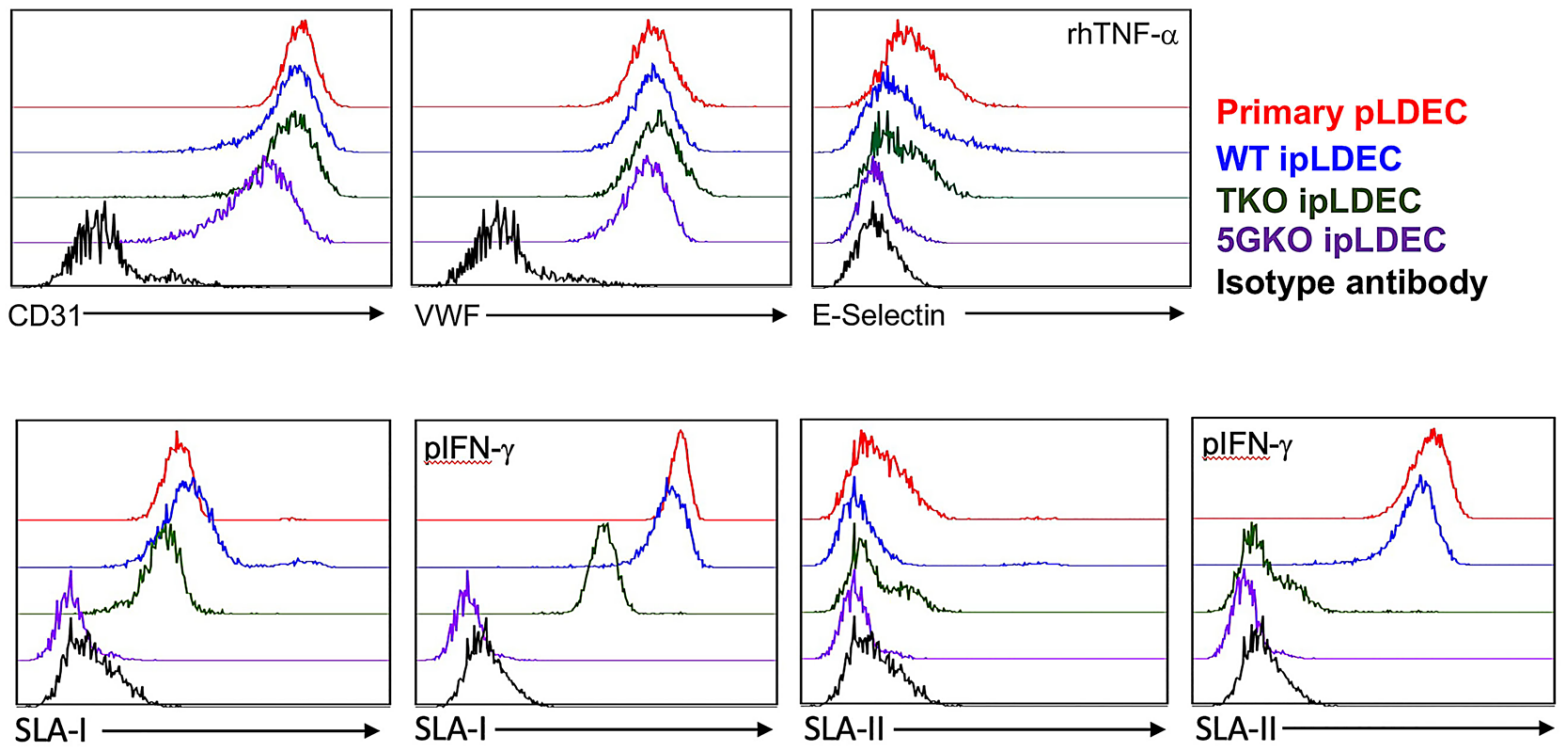
Characterization of primary pLDEC and immortalized porcine liver-derived endothelial cells (ipLDEC). Porcine cells were analyzed for endothelial cell markers, including cell surface expression of CD31, intracellular expression of von Willebrand factor (VWF), and E-selectin (rhTNF-α stimulation) by flow cytometry. SLA-I and SLA-II expression in porcine cells before and after porcine IFN-γ stimulation (pIFN-γ) were examined by flow cytometric analysis using specific antibodies. Primary pLDEC (passage 15–20) and ipLDEC (passage 20–30 after immortalization) were used for characterization.

Primary pLDEC passages between 15 and 20 and WT, TKO, and 5GKO ipLDEC passages between 20 and 30 after immortalization were used for endothelial cell characterization. Flow cytometric analysis showed that WT, TKO, and 5GKO ipLDEC expressed endothelial cell markers CD31 and von Willebrand factor (VWF) as primary pLDEC (Fig. 4). Cells were stimulated with recombinant human TNF-α (rhTNF-α) at 20 ng/mL for 16 h, E-selectin expression was detected in pLDEC and WT ipLDEC but not in TKO and 5GKO ipLDEC. As expected, SLA-I molecules were found in pLDEC, WT, and TKO ipLDEC, but not in 5GKO. Upon porcine IFN-γ (pIFN-γ) stimulation, SLA-I expression was dramatically upregulated in pLDEC, WT, and TKO ipLDEC. No SLA-I was detected in 5GKO cells after pIFN-γ stimulation. SLA-II expression were not found in all porcine cell lines but were detected in pLDEC and WT ipLDEC after pIFN-γ stimulation (Fig. 4). Together, expression of endothelial cell markers including CD31, VWF, and E-selectin along with de novo expression of SLA-II molecules and increased surface expression of SLA-I molecules responding to pIFN-γ stimulation, indicated that WT ipLDEC retained endothelial cell property as primary pLDEC. TKO and 5GKO ipLDEC exhibited partial endothelial cell properties by expressing CD31 and VWF but fail to express E-selectin and SLA-II molecules after cytokine stimulation. CD31, VWF, and SLA-I were frequently checked before all functional experiments, the expression levels were consistent.

### Reduced human antibody reactivity to TKO and 5GKO ipLDEC

**Figure 5:**
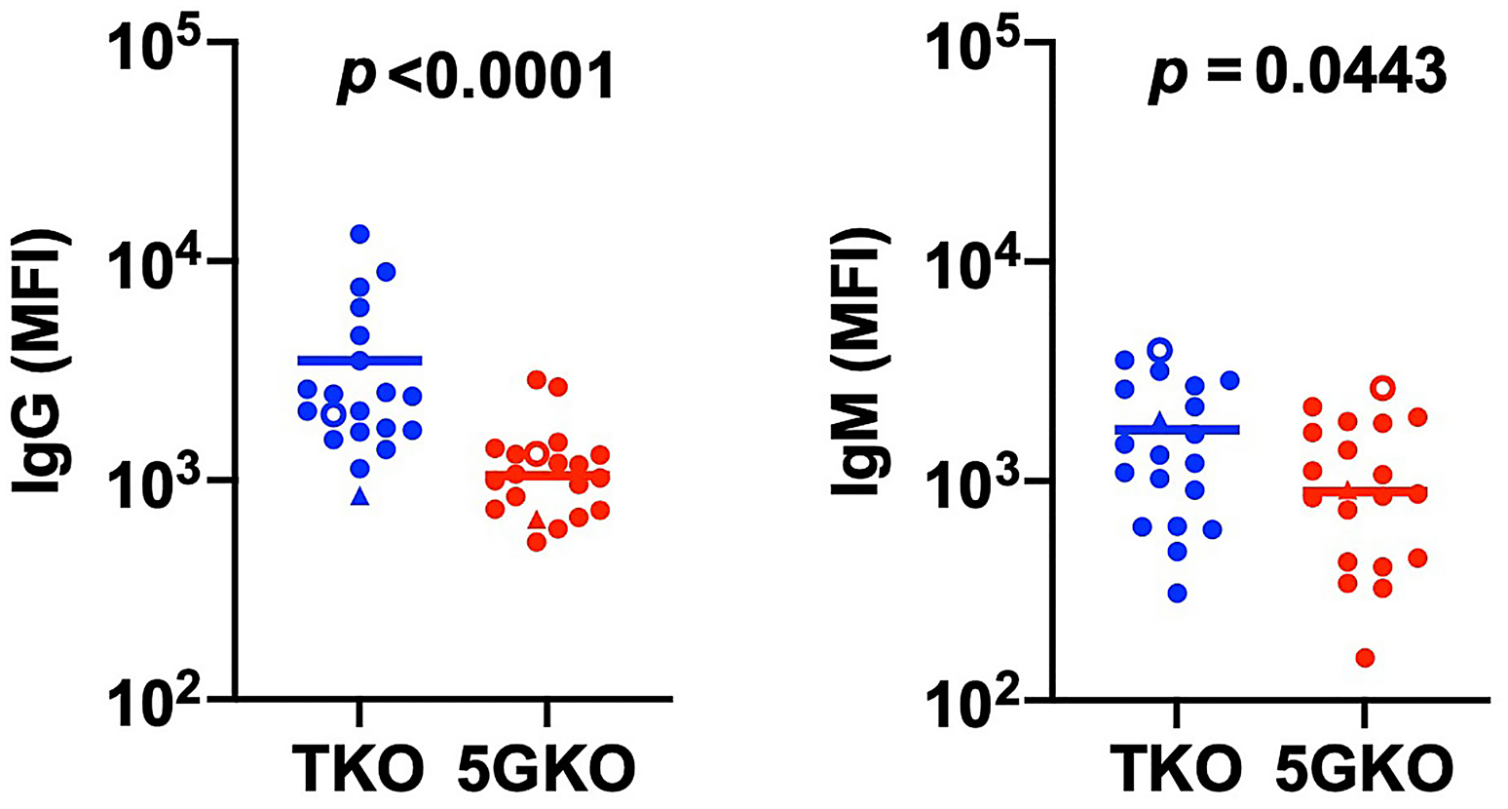
Comparison of human serum antibody binding to TKO and 5GKO ipLDEC lines. Human sera from 20 patients on the kidney transplant wait-list were incubated with TKO (*GGTA1/CMAH/β4galNT2*) or 5GKO (*GGTA1/CMAH/ β4galNT2/SLA-I α chain/B2M*) ipLDEC. Subsequently, fluorescent conjugated anti-human IgG or IgM was used to evaluate human IgG or IgM binding to the cells, with mean fluorescence intensity (MFI). Mann–Whitney test was used to analyze the differences between TKO and 5GKO ipLDEC lines. Human serum IgG exhibited significantly decreased binding to 5GKO ipLDEC compared to TKO ipLDEC (*p* < 0.0001). Human serum IgM binding to 5GKO ipLDEC was also significantly reduced compared to TKO ipLDEC, (*p* = 0.0443). Unfilled dots and triangles represented low PRA (< 10%) serum samples (n = 2). All other serum samples (n = 18) shown in dots were from individuals with high PRA (> 90%).

Previous studies indicated that some human HLA class I antibodies cross-react with swine SLA-I molecules^16,25,26^. Elimination of SLA-I on porcine cells may diminish HLA class I antibody binding to prevent antibody-mediated rejection. We compared human antibody reactivity to TKO and 5GKO ipLDEC lines. Human serum samples were from 20 patients on the kidney transplant wait-list with panel-reactive antibody (PRA) in the range of low (< 10%, n = 2) to high (> 90%, n = 18). Preliminary assessment of data normality was performed and Mann–Whitney test was selected for the statistical analysis. Human IgG binding to 5GKO ipLDEC was significantly reduced compared to human IgG binding to TKO ipLDEC (*p* < 0.0001). Human IgM reactivity to 5GKO ipLDEC compared to TKO ipLDEC was also statistically decreased (*p* = 0.0443) (Fig. 5). In addition, we compared human antibody binding to WT, TKO, and 5GKO ipLDEC. Both TKO and 5GKO ipLDEC exhibited reduced reactivity to human IgG and IgM compared to WT ipLDEC, as expected (Supplementary Fig. S2).

### Activation of human natural killer (NK) cells by WT, TKO, and 5GKO ipLDEC

NK cells can directly recognize and lyse target cells through their receptors interacting with the ligands on target cells^18^. CD107a or lysosome-associated membrane protein-1 (LAMP-1) is a marker of immune cell activation and cytotoxic degranulation.

A previous study indicated that CD107a surface expression has a direct correlation with NK cell cytotoxic activity and cytokine secretion, which is a reliable marker for evaluation of activation of immune cell cytotoxic responses against stimuli such as xenogeneic cells^27^. Various effector:target (E:T) ratio (1:1, 2:1, 5:1, 10:1, 25:1, 50:1, 100:1) had been tested and E:T ratio of 10:1 was found an optimal for CD107a expression as well as cytokine secretion using multi-parameter flow cytometry to monitor NK activation^27^.

**Figure 6:**
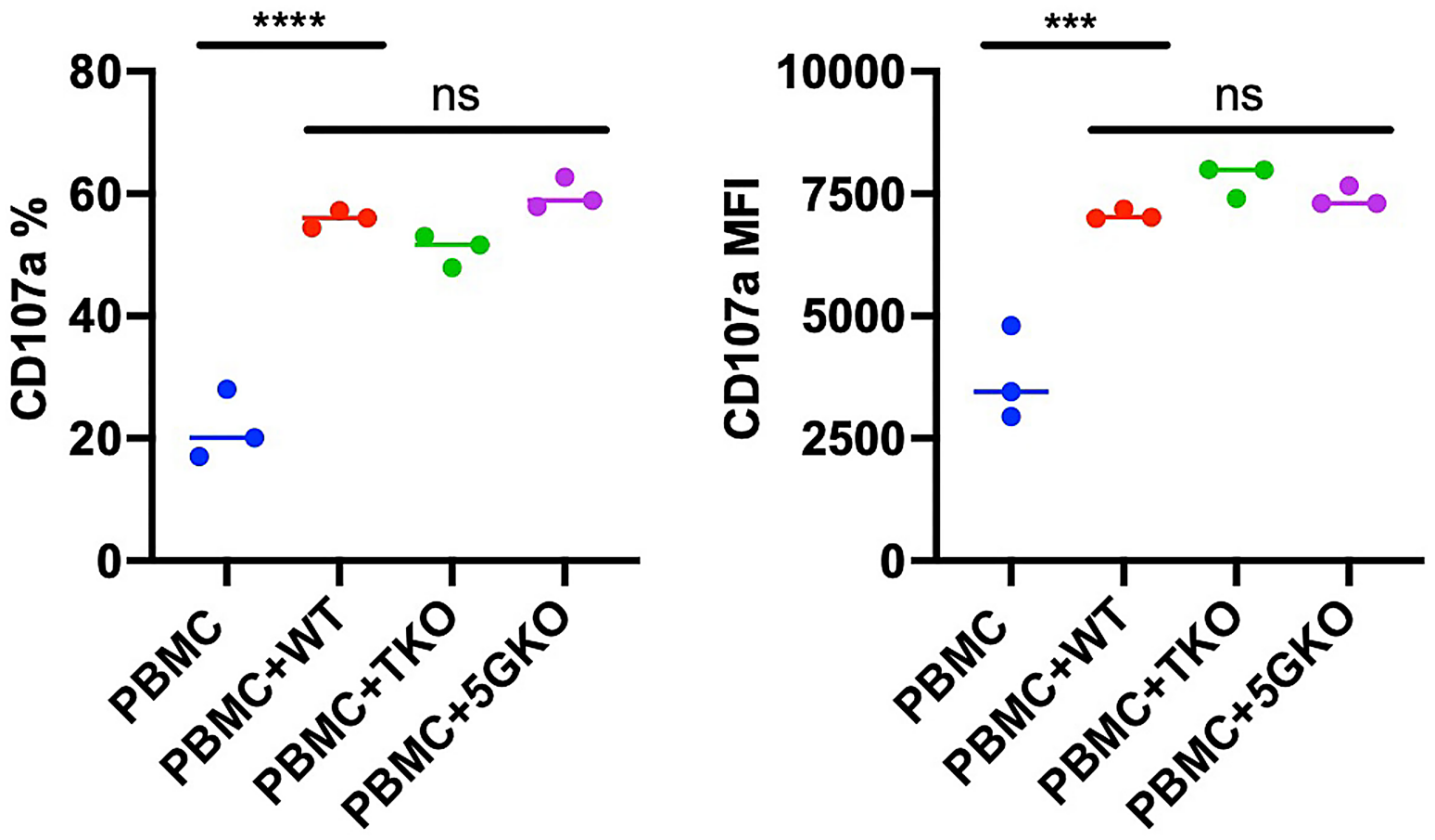
Human NK cell degranulation following porcine cell stimulation. Human PBMCs from three donors were treated with human IL-2 (20 ng/mL) for 5 days to expand NK cells, then cocultured with WT, TKO, and 5GKO ipLDEC for 2 h. Cells were stained with antibodies to CD45, CD3, CD56, and CD107a. Flow cytometry analysis was gated on NK cells (CD3^-^CD56^+^) and monitored for the percentage of CD107a positive cells (**left**) and surface expression of CD107a (MFI) (**right**). Statistical significance was calculated by one-way ANOVA, Tukey’s multiple comparisons test (*ns* not significant; ***p < 0.001; ****p < 0.0001).

Human PBMCs were isolated from the ‘buffycoats’ of three individuals and pre-treated with recombinant human IL-2 (rhIL-2) at 41.2 U/mL to expand NK effector cells. Human PBMCs were co-cultured with WT, TKO, and 5GKO ipLDEC lines at E:T ratio of 10:1 for 2 h, and harvested for cell surface marker staining using fluorochrome-conjugated antibodies against CD45, CD3, CD56, and CD107a. Human NK cell degranulation to porcine cell stimulation was measured by the percentage of CD107a-positive cells and surface expression of CD107a in the CD3^-^CD56^+^ cell population. There were no significant differences in either percentage or mean fluorescent intensity (MFI) of CD107a among WT, TKO, and 5GKO ipLDEC lines (Fig. 6), suggesting that elimination of carbohydrate xenoantigens (αGal, Neu5Gc, and Sda) and SLA-I had no effects in human NK cell activation via ligand-receptor interactions.

## Discussion

Pig-to-NHPs pre-clinical XTx trials are essential for the clinical application of XTx. However, different GM pigs may be required for testing in NHPs and humans (due to species disparity between humans and NHPs) in order to prevent immune response, such as antibody mediated rejection^13^. In vitro evaluation of the human immune responses to porcine cells with various genetic modifications may provide a straightforward approach to quickly identify the benefit of combinational genetic modifications.

In the present study, TKO (*GGTA1*, *CMAH*, and *β4galNT2*) and 5GKO (TKO, *SLA-I α chain* and *β2-microglobulin*) were generated in immortalized porcine liver derived endothelial cell line using CRISPR/Cas9. Both TKO and 5GKO ipLDEC exhibited reduced reactivity to human antibody binding compared to WT cells. Our results indicated that human antibody reactivity to TKO ipLDEC is consistent and comparable to human antibody reactivity to PBMCs isolated from TKO pig^9^. A previous study indicated that 69% of 820 renal transplant waitlist patients had antibodies against PBMC from TKO pigs, some of antibodies were HLA class I antibodies cross-reacting with SLA-I molecules^16^. Removal of SLA-I in a TKO pig is necessary for highly sensitized patients who have little chance to obtain a matched renal allograft but may benefit from a pig organ that does not express SLA-I, αGal, Neu5Gc, and Sda xenoantigens. Fischer et al. reported that porcine fibroblasts derived from pigs deficient in SLA-I, αGal, Neu5Gc, and Sda xenoantigens, exhibited further decreased reactivity to human antibody binding^10^, which is consistent with the results of our study. 5GKO ipLDEC line has significantly reduced reactivity to human IgG and IgM binding, compared to the TKO ipLDEC line. Both TKO and 5GKO ipLDEC are valuable reagents and ready to use in a crossmatch assay, avoiding the generation and maintenance of TKO and 5GKO pigs for cell isolation.

SLA-I-deficient porcine cells were generated by the disruption of *SLA-I α chains* and *B2M* genes. *SLA-I* genes (*α chains*) are highly polymorphic with 227 alleles designated to date (https://www.ebi.ac.uk/ipd/mhc/group/SLA/). The *SLA-I* gene cluster contains three constitutively expressed classical genes, *SLA-1*, *SLA-2*, and *SLA-3*. Cas9 and two gRNAs targeting the common regions of multiple alleles in *SLA-1*, *SLA-2*, and *SLA-3* completely abolished SLA-I expression. *B2M* was subsequently disrupted, and the mutation was confirmed by Sanger sequencing. Since we plan to express human HLA class I molecules in the 5GKO ipLDEC line in the future, removal of both SLA-I α chains and B2M is necessary to prevent the formation of SLA-I or HLA-class I hybrids. We found that SLA-I α chain can pair with human B2M to rescue SLA-I expression (unpublished data).

WT ipLDEC and primary pLDEC had similar morphology and endothelial cell features. WT ipLDEC retained endothelial cell property by expressing endothelial cell markers such as CD31, VWF, and E-selectin with rhTNF-α stimulation. WT ipLDEC expressed elevated SLA-I and SLA-II upon pIFN-γ stimulation. TKO and 5GKO expressed CD31 and VWF but not E-selectin and SLA-II after cytokine stimulation. Moderate increased SLA-I expression was found in TKO with pIFN-γ stimulation. There was no SLA-I expression in 5GKO as expected. Absence of E-selectin and SLA-II in TKO and 5GKO was unknown and whether it was associated with genetic modifications needs to be investigated. It would be interesting to compare human cellular response to cytokine activated WT, TKO, and 5GKO ipLDEC in the future study.

It is important to investigate whether the removal of three xenoantigens (αGal, Neu5Gc, Sda) on pig cells affects human NK cell-mediated direct cytotoxicity. Our data showed, for the first time, that the depletion of three xenoantigens had no effects on direct human NK activation. Human NK cells are one of the components of the innate immune system and are involved in xenograft rejection. NK cell infiltration was found in pig organs perfused with human blood^28,29^ and in pig-to-NHP xenografts^30,31^. Previous studies indicated that elimination of αGal on porcine endothelial cells had no effects on direct xenogeneic lysis mediated by human NK cells or NK cell line NK92^32,33^.

In an allogeneic setting, self-MHC class I is a signal for NK cell tolerance. Whether SLA-I can serve as an inhibitory ligand, like its counterpart HLA class I, to human NK cell activation is uncertain. Sullivan et al. reported that SLA-I is unable to efficiently transmit inhibitory signals to human NK cells because amino acid residues critical for the binding to human NK cell inhibitory receptors are altered in SLA-I when compared to HLA class I^34^. Another study showed that the induction of SLA-I expression on porcine endothelium by tumor necrosis factor-alpha (TNF-α) reduced lysis by human NK cells^35^. We compared the human NK cell response to SLA-I-deficient porcine cells and SLA-I^+^ porcine cells. Our results showed that human NK cells became activated by WT, TKO, and 5GKO ipLDEC stimulation. There was no significant difference among three groups with the limitation of small sample numbers. SLA-I expression had no effects on triggering NK cell activation. Hein et al. reported that B2M^low^ porcine fibroblasts were not protected from human NK mediated cytotoxicity^36^, which was consistent with our findings. Taken together, these results indicated that SLA-I is *not* an inhibitory ligand for human NK cells.

If antibody-mediated immune rejection can be controlled, the prevention of the cellular immune rejection will become the main focus in XTx research. In pig-to-NHP preclinical XTx trials, various immunosuppressants have been used to prevent the cellular immune response, which are often either not clinically-applicable or not practical due to deleterious side-effects. Development of a novel way to induce NK cell immune tolerance and improve human-pig compatibility is needed to speed XTx application. Manipulation of NK ligands on porcine cells, including removal of activating ligands and introducing inhibitory ligands, may protect xenografts from immune cell-mediated cytotoxicity. Establishment of WT, TKO, and 5GKO ipLDEC lines provides valuable in vitro models for further genetic engineering that may help to mitigate human cellular immune responses, which will ultimately contribute to long-term survival of pig xenografts in humans.

## Methods

### Immortalization of pLDEC

pLDEC (passage 12) were immortalized by SV40 T antigen and hTERT lentiviral treatment, according to the manufacturer’s instruction (Applied Biological Materials, Richmond, BC, Canada). After immortalization, WT ipLDECs were kept in culture for various genetic engineering and functional assays.

### Live cell imaging

The images of pLDEC (passage 12) and ipLDEC (passage 25 after immortalization) were taken by Leica DMi1 Inverted Microscope (Leica Microsystems, Wetzlar, Germany).

### Knockout of multiple-gene encoding xenoantigens

In order to delete multiple porcine genes using CRISPR/Cas9, the gRNAs targeting *GGTA1*, *CMAH*, *β4galNT2*, *SLA-I α chain*, and *B2M* were designed and cloned to CRISPR/Cas9 vector pX330 and/or PX458, respectively. pX330-U6-Chimeric_BB-CBh-hSpCas9 and pSpCas9(BB)-2A-GFP (PX458) were gifts from Dr. Feng Zhang (Addgene plasmid #42230 and #48138). The sequences of gRNA target and gRNA oligos are listed in Table 1. Co-transfection of ipLDEC with multiple gRNAs targeting *GGTA1*, *CMAH*, and *β4galNT2* genes was performed as previously described^9^. Briefly, 10^6^ of ipLDEC were mixed with 2 μg of each plasmid and electroporated at 1300 V, 30 ms, 1 pulse using Neon Transfection System (Thermo Fisher Scientific, Waltham, MA). After 48 h, transfected cells were enriched by flow sorting for GFP-positive cells, and sequentially selected for the absence of αGal and Sda, and reduction of Neu5Gc, by Dynabeads isolation and flow cytometry cell sorting^9,11,37^. The reagents used for selection were Isolectin B4 (IB4)-biotin (Enzo Life Sciences, Farmingdale, NY), Dynabeads Biotin Binder (Thermo Fisher Scientific), Dolichos biflorus Agglutinin (DBA)-FITC (Vector Laboratories, Burlingame, CA), and chicken anti-Neu5Gc antibody-Alexa Fluor 488 (BioLegend, San Diego, CA). The mutation region of each gene was confirmed by PCR amplification and Sanger sequencing using specific primers (Table 2). Triple gene knockout (TKO; *GGTA1/CMAH/ β4galNT2*) ipLDEC were generated.

Due to the high polymorphism of *SLA-I α chain*, several gRNAs targeting the common sequences of *SLA-1*, *SLA-2*, and *SLA-3* multiple alleles were designed and tested for efficiency. TKO ipLDEC were transfected with two selected gRNAs targeting *SLA-I α chains*. After 48 h, transfected cells were stained with mouse anti-SLA class I-FITC (Bio-Rad Laboratories, Hercules, CA) and SLA-I-deficient cells were selected by flow cytometry cell sorting. Then this knockout cell line (*GGTA1/CMAH/ β4GalNT2/SLA-I α chain*) was transfected with the gRNA targeting *B2M* gene. B2M mutant cells were selected by a single-clone screening approach using PCR and Sanger DNA sequencing. 5-gene knockout (5GKO; *GGTA1/CMAH/ β4GalNT2/SLA-I α chain/B2M*) ipLDECs were created.

### Characterization of primary pLDEC and ipLDEC

Primary porcine liver-derived cells, WT, TKO, and 5GKO immortalized cells were cultured in media (α‐MEM:EGM‐MV 3:1) (Invitrogen/Lonza, Switzerland) supplemented with 10% FBS (Hyclone, Logan, UT), 10% horse serum (Invitrogen, Carlsbad, CA), 12 mM HEPES, and 1% pen/strep (Invitrogen), as described in our previous study^22^. Briefly, liver tissue was harvested from mature female crossbred domestic pigs between the ages of 8 and 10 months at a local surgical unit. All the animals used and procedures performed on animals were approved by Indiana University School of Medicine Institutional Animal Care and Use Committee (IACUC). Expression of endothelial cell markers as well as SLA molecules were examined by flow cytometric analysis using specific antibodies. CD31 expression was monitored by staining cells with FITC-conjugated mouse anti-pig CD31 antibody (Bio-Rad Laboratories). Intracellular VWF expression was examined by FITC-conjugated sheep anti-Von Willebrand Factor antibody (Abcam, Cambridge, MA) after fixation and permeabilization of cells. To examine E-selectin expression, cells were activated with recombinant human TNF-α (rhTNF-α) at 20 ng/mL for 16 h, and then stained with FITC-conjugated mouse anti-human CD62E/CD62P antibody (Bio-Rad Laboratories). This antibody can cross-react with pig E-selectin^38^. To examine SLA-I and SLA-II molecules expression, cells were treated with or without porcine IFN-γ (pIFN-γ) (R&D Systems, Minneapolis, MN) at 10 ng/mL for 60 h, then stained with mouse anti-pig SLA-I-FITC antibody (Bio-Rad Laboratories) and/or mouse anti-pig SLA-II DR-FITC antibody (Bio-Rad Laboratories). Isotype antibodies were used as controls. After staining, cells were washed and subsequently analyzed using a LSR4 flow cytometer (BD Bioscience, Sam Jose, CA). Flow data were analyzed using FlowJo v10 software (BD Bioscience).

### Human antibody binding to TKO and 5GKO porcine cells

Human sera range from 0.5% to 50% had been titrated in human antibody binding to pig endothelial cells in our previous study^39^. An optimal concentration 25% of sera was used in this study. A human antibody binding assay was performed as previously described^11,16^. Briefly, 2 × 10^5^ TKO or 5GKO ipLDECs were washed and incubated with 25% heat-inactivated human serum in EX-CELL 610-HSF Serum-Free Medium (Sigma, St. Louis, MO) with 0.1% sodium azide for 1 h at 4 °C. Cells were washed three times with EX-CELL 610-HSF Serum-Free Medium, and stained with donkey anti-human IgG Alexa Fluor 488 (Jackson ImmunoResearch Laboratories, West Grove, PA) or donkey anti-human IgM Alexa Fluor 647 (Jackson ImmunoResearch Laboratories) for 30 min at 4 °C. Cells were washed, fixed with 2% paraformaldehyde (PFA), and subsequently analyzed using a LSR4 flow cytometer (BD Biosciences, San Jose, CA). TKO and 5GKO ipLDECs stained with donkey anti-human IgG Alexa Fluor 488 or donkey anti-human IgM Alexa Fluor 647 were used as negative controls. Flow data were analyzed using FlowJo v10 software (BD Biosciences). Human IgG or IgM binding to TKO and 5GKO ipLDEC were represented by mean fluorescence intensity (MFI) subtracting negative control MFI. In addition, human antibody binding to WT, TKO, and 5GKO ipLDEC were examined to ensure the reduced antigenicity in TKO and 5GKO ipLDEC compared to WT ipLDEC.

### Human NK cell degranulation

Commercially available buffy coats were acquired from Versiti Indiana Blood Center. Human peripheral blood mononuclear cells (PBMC) were isolated using Ficoll-Paque Plus (GE-Healthcare, Pittsburgh, PA) gradient centrifugation and cultured in RPMI1640 with 10% FBS, 1% pen/strep, and 20 ng/mL rhIL-2 (~ 41.2 U/mL, BioLegend) at 37 °C in a CO_2_ incubator for 5 days. 5 × 10^4^ WT, TKO, and 5GKO ipLDECs were plated in 48-well plates in duplicate or triplicate one day ahead of co-culture. 5 × 10^5^ PBMC were added to porcine cells at an E:T ratio of 10:1, and co-cultured for 2 h at 37 °C in a CO_2_ incubator. Then cells were collected and stained with fluorochrome-conjugated antibodies against human CD45, CD3, CD56, CD107a (BioLegend), and Invitrogen eBioscience Fixable Viability Dye eFluor 780 (Thermo Fisher Scientific). Stained cells were fixed with 2% PFA and subsequently analyzed using a LSR4 flow cytometer (BD Biosciences). After pre-gating on CD45^+^ live singlets, NK cell degranulation activity was assessed based on percentage and mean fluorescence intensity (MFI) of CD107a in a CD3^-^CD56^+^ cell population. Flow data were analyzed using FlowJo v10 software (BD Biosciences).

### Statistical analysis

Statistical analyses were performed using GraphPad Prism 9 software (GraphPad Software, San Diego, CA). Normality test was used to assess data distribution. Mann–Whitney test was used to analyze the differences between the two groups. One-way ANOVA, Tukey’s multiple comparisons test was used to analyze the differences among multiple groups.

## Supplementary Information

Supplementary Information.
