# Publisher Correction: Genetic engineering of porcine endothelial cell lines for evaluation of human-to-pig xenoreactive immune responses

**DOI:** 10.1038/s41598-021-96406-4

**Published:** 2021-08-16

**Authors:** Ping Li, Julia R. Walsh, Kevin Lopez, Abdulkadir Isidan, Wenjun Zhang, Angela M. Chen, William C. Goggins, Nancy G. Higgins, Jianyun Liu, Randy R. Brutkiewicz, Lester J. Smith, Hidetaka Hara, David K. C. Cooper, Burcin Ekser

**Affiliations:** 1 Division of Transplant Surgery, Department of Surgery, Indiana University School of Medicine, Indianapolis, IN USA; 2 Indiana University Health, Indianapolis, IN USA; 3 Department of Microbiology and Immunology, Indiana University School of Medicine, Indianapolis, IN USA; 4 Radiology and Imaging Sciences, Indiana University School of Medicine, Indianapolis, IN USA; 5 3D Bioprinting Core, Indiana University School of Medicine, Indianapolis, IN USA; 6 Xenotransplantation Program, Department of Surgery, University of Birmingham at Alabama, Birmingham, AL USA; 7 Present Address: Weldon School of Biomedical Engineering, West Lafayette, IN USA

Correction to: *Scientific Reports* 10.1038/s41598-021-92543-y, published online 23 June 2021

The original version of this Article contained an error in the Abstract and in the Results section where the beta symbol did not display correctly.

This error has now been corrected in the PDF version of the Article; the HTML version was correct from the time of publication.

