# Supplementary material for: Genetic engineering of porcine endothelial cell lines for evaluation of human-to-pig xenoreactive immune responses
Source: Sci Rep. 2021 Jun 23;11:13131. doi: 10.1038/s41598-021-92543-y (PMC8222275; doi:10.1038/s41598-021-92543-y)
Supplement: Supplementary file 1 — Supplementary Information. [file 41598_2021_92543_MOESM1_ESM.pdf]

Genetic engineering of porcine endothelial cell lines for evaluation of human-to-pig xenoreactive immune responses

Ping Li, PhD[1*], Julia R. Walsh, BS[1#], Kevin Lopez, MD[1], Abdulkadir Isidan, MD[1], Wenjun Zhang, PhD[1], Angela M. Chen, MD, MS[1], William C. Goggins, BS[1], Nancy G. Higgins, BS[2], Jianyun Liu, PhD[3], Randy R. Brutkiewicz, PhD[3], Lester J. Smith, PhD[4,5], Hidetaka Hara, MD, PhD[6], David K.C. Cooper, MD, PhD[6], Burcin Ekser, MD, PhD[1*]

[1]Division of Transplant Surgery, Department of Surgery, Indiana University School of Medicine, Indianapolis, Indiana, United States; [2]Indiana University Health, Indianapolis, Indiana; [3]Department of Microbiology and Immunology, Indiana University School of Medicine, Indianapolis, Indiana, United States; [4]Radiology and Imaging Sciences, Indiana University School of Medicine, Indianapolis, Indiana, United States; [5]3D Bioprinting Core, Indiana University School of Medicine, Indianapolis, Indiana, United States; [6]Xenotransplantation Program, Department of Surgery, University of Birmingham at Alabama, Birmingham, Alabama, United States; [#]Current address: Weldon School of Biomedical Engineering, West Lafayette, Indiana

<u>Address for correspondence:</u>
*Ping Li, PhD
*Burcin Ekser, MD, PhD
Division of Transplant Surgery, Department of Surgery
Indiana University School of Medicine
980 W. Walnut Street, R3-C634
Indianapolis, IN 46202
Tel: 317-274-0797
Email: pili@iupui.edu; bekser@iupui.edu

Word count: Abstract 184; Main text 2961; Tables 2, Figures 6; Supplementary Figures 2

Supplementary Information

Deletion of the first nucleotide of *CMAH* exon 4 resulted in a frame shift. Mutated *CMAH gene* was translated and aligned with CMAH protein using MacVector 18.0 software (MacVector, Apex, NC) (Supplementary Fig. S1).

Human serum samples were from three patients on the kidney transplant wait-list with high PRA (>90%). Human IgG and IgM reactivity to WT, TKO, and 5GKO ipLDEC were examined by flow cytometry based antibody binding assay. Both TKO and 5GKO ipLDEC exhibited reduced reactivity to human IgG and IgM compared to WT ipLDEC (Supplementary Fig. S2).

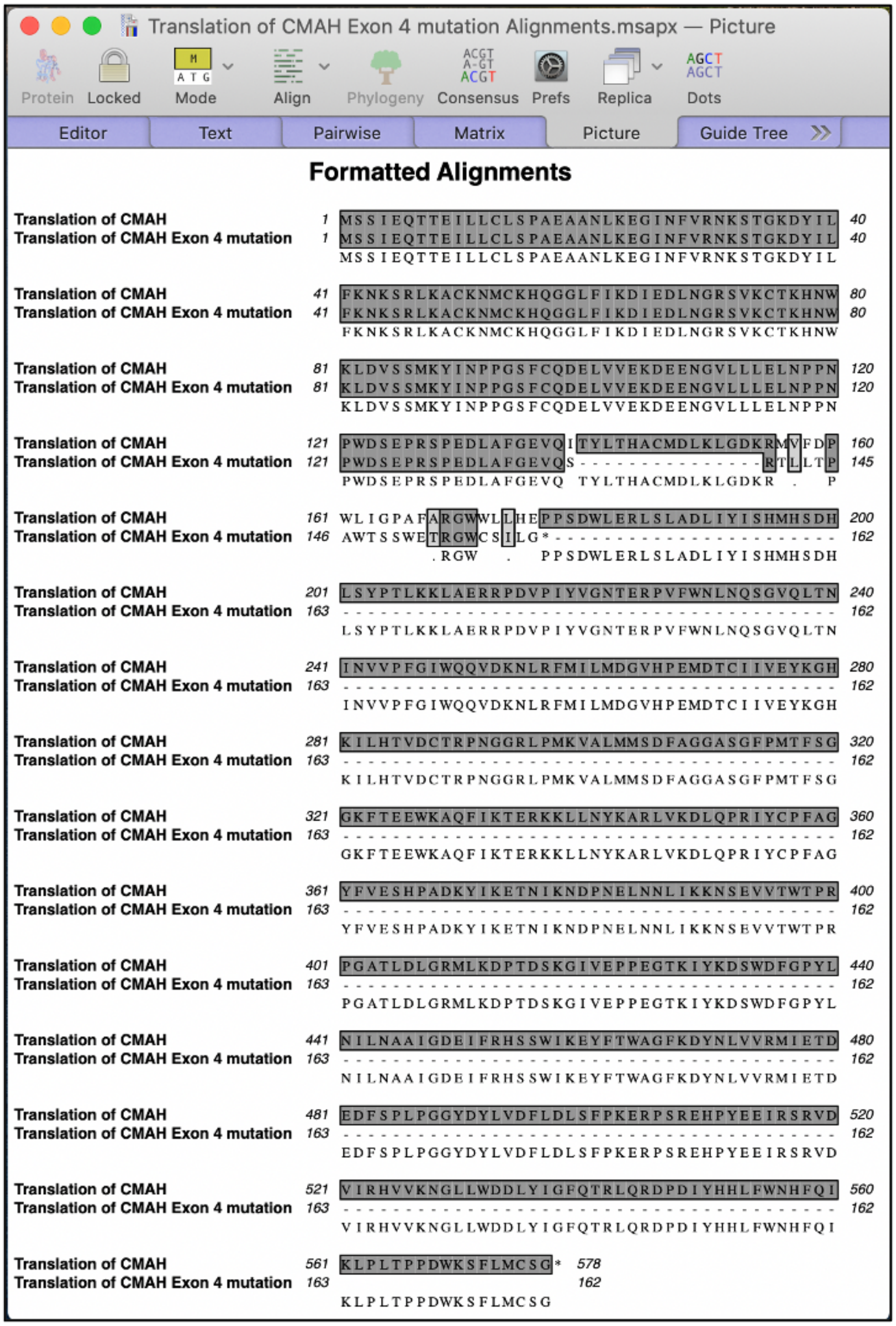

Supplementary Fig. S1. Alignment of CMAH protein and mutated *CMAH* gene encoded truncated polypeptide. Functional CMAH has 578 amino acids while CMAH mutant has 162 amino acids.

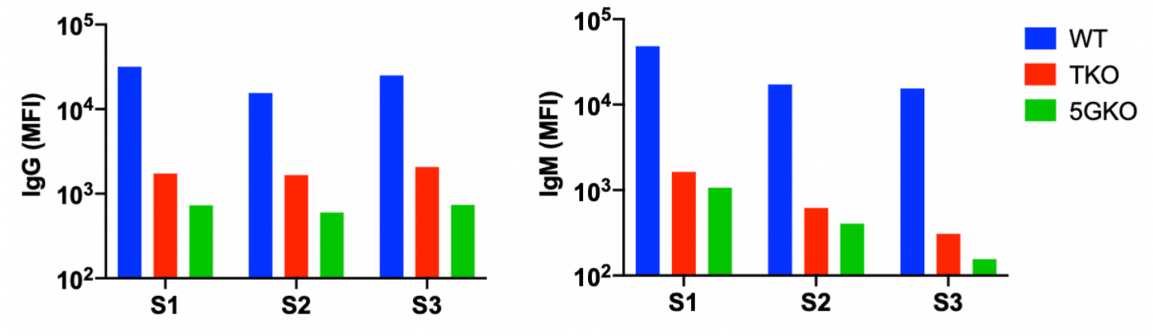

Supplementary Fig. S2. Comparison of human antibody binding to WT, TKO, and 5GKO ipLDEC.
